# Improving Nutritional and Antioxidant Properties of Dried Noodles Through Utilization of *Acanthus ilicifolius* Leaf Flour for Sustainable Food Development

**DOI:** 10.1155/tswj/4984469

**Published:** 2026-07-29

**Authors:** Diana Nur Afifah, Katarina Aletta Sahara, Bagas Naufal Pudyastungkara, Laurensia Aldora Bachtiar, Denny Nugroho Sugianto, Dessy Ariyanti, Bulan Prabawani, Wiwandari Handayani

**Affiliations:** ^1^ Department of Nutrition Science, Faculty of Medicine, Universitas Diponegoro, Semarang, Central Java, Indonesia, undip.ac.id; ^2^ SDGs Center, Universitas Diponegoro, Semarang, Central Java, Indonesia, undip.ac.id; ^3^ Laboratory of Sustainable Diets and Biodiversity, Integrated Laboratory for Research and Services, Universitas Diponegoro, Semarang, Central Java, Indonesia, undip.ac.id; ^4^ Department of Oceanography, Faculty of Fisheries and Marine Science, Universitas Diponegoro, Semarang, Central Java, Indonesia, undip.ac.id; ^5^ Department of Chemical Engineering, Faculty of Engineering, Universitas Diponegoro, Semarang, Central Java, Indonesia, undip.ac.id; ^6^ Department of Business Administration, Faculty of Social and Political Sciences, Universitas Diponegoro, Semarang, Central Java, Indonesia, undip.ac.id; ^7^ Department of Urban and Regional Planning, Faculty of Engineering, Universitas Diponegoro, Semarang, Central Java, Indonesia, undip.ac.id

## Abstract

Instant dried noodles are popular in Asia but are generally low in fiber and antioxidant content. *Acanthus ilicifolius* leaf flour, which is rich in bioactive compounds, has the potential to be used as a functional ingredient to enhance nutritional quality. Four noodle formulations were prepared by substituting wheat flour (WF) with *Acanthus ilicifolius* flour (AF): F0 (100% WF), F1 (92% WF: 8% AF), F2 (84% WF: 16% AF), and F3 (76% WF: 24% AF). Proximate composition, dietary fiber (AOAC 1995), and antioxidant activity (DPPH assay) were analyzed, along with hedonic and Just‐About‐Right sensory tests. Substitution significantly reduced the carbohydrate and energy content but increased the ash, protein, crude fiber, and total dietary fiber content. The fat content remained stable across all formulations. The antioxidant activity improved markedly, as the IC_50_ values decreased from 25,167.79 ppm (F0) to 4324.72 ppm (F3). Sensory evaluation revealed reduced acceptability at higher substitution levels, although the texture remained unaffected. Partial substitution with *A. ilicifolius* leaf flour enhances the nutritional and functional properties of dried noodles. An 8% substitution (F1) appears to be optimal, balancing improved health benefits with consumer acceptance.

## 1. Introduction

The increasing prevalence of noncommunicable diseases (NCDs), such as obesity, cardiovascular disease, and Type 2 diabetes mellitus, has been strongly associated with dietary patterns characterized by excessive refined carbohydrate intake and inadequate dietary fiber consumption [1]. Instant dried noodles, a staple food widely consumed in Asia including Indonesia, are typically high in refined carbohydrates but deficient in fiber and antioxidants, limiting their nutritional contribution despite their convenience and affordability [2]. Addressing these nutritional gaps requires the reformulation of staple foods into functional products with enhanced health benefits.

One promising strategy involves partially substituting wheat flour (WF) with mangrove‐derived flours, such as those obtained from *Acanthus ilicifolius* leaves. This underutilized local resource is rich in bioactive compounds, including flavonoids, tannins, and polyphenols, which exhibit strong antioxidant, antimicrobial, and anti‐inflammatory properties [3]. Such innovations are consistent with global development priorities, particularly Sustainable Development Goal (SDG) 2, which promotes improved nutrition and food security through diverse and sustainable food sources; SDG 3, which emphasizes reducing the burden of NCDs and promoting overall health and well‐being; and SDG 15, which supports the sustainable use of terrestrial ecosystems, including mangrove biodiversity, for environmental and community benefits.

Beyond noodles, *A. ilicifolius* has been studied for various food and health applications. Past research demonstrated that *A. ilicifolius* leaf extract can be formulated into a functional beverage with significant antibacterial activity and high levels of phenolic compounds [4]. In vitro research has further revealed its potential antidiabetic activity through inhibition of *α*‐amylase and *α*‐glucosidase, enzymes responsible for carbohydrate metabolism, thereby supporting glycemic regulation [5]. Additionally, past study also reported the gastroprotective and antiulcer effects of *A. ilicifolius* methanolic extracts, which significantly reduced gastric lesions and oxidative stress markers in experimental models [6]. Collectively, these studies highlight the potential of *A. ilicifolius* not only as a functional ingredient for staple foods but also as a versatile source of health‐promoting compounds with broad applications in human nutrition.

Building on this evidence, the present study is aimed at developing and characterizing functional dried noodles by partially substituting WF with *A. ilicifolius* leaf flour. Quality evaluation encompasses proximate composition, dietary fiber content, antioxidant activity, and sensory properties to determine the feasibility of incorporating this mangrove species into widely consumed food products.

## 2. Materials and Methods

### 2.1. *A. ilicifolius*

The *A. ilicifolius* leaf flour used in this study was obtained from coastal areas in Demak Regency, Central Java, Indonesia, and supplied in a ready‐to‐use form by Bina Agro Mandiri. Fresh *A. ilicifolius* leaves were washed, air‐dried for 2 h, and then oven‐dried at 60°C for 24 h (optimal temperature for phytochemical retention). Dried leaves were milled using a hammer mill and sieved through a 60‐mesh sieve to obtain a fine leaf flour. Laboratory analysis showed that the flour contained 0.09% tannins, 67.88% insoluble dietary fiber, 3.31% soluble dietary fiber, 71.18% total dietary fiber, 38.35 ppm hydrogen cyanide (HCN), and 615.81 ppm antioxidant activity. Tannin content was determined using a spectrophotometry method adapted from Chanwitheesuk (2004) [7]. Insoluble, soluble, and total dietary fibers were determined using the AOAC (1995) multienzyme enzymatic‐gravimetric method [8]. HCN analysis procedure was determined using the alkaline picrate method (Procedures for Food and Agricultural Materials Analysis, 4th Edition, Modified Spectrophotometry) [9]. Antioxidant activity was measured using the DPPH radical‐scavenging assay, as adapted from Molyneux [10]. The high dietary fiber content, particularly insoluble fiber, highlights its potential to improve digestive health and reduce the risk of NCDs, whereas the presence of tannins and phenolic compounds supports strong antioxidant properties.

Although the flour contained HCN, the level (38.35 ppm) remained below the maximum safe limit of 50 ppm, as established by the World Health Organization and the Indonesian National Standard (SNI 7387: 2009) [11, 12]. This confirms the safety of *A. ilicifolius* leaf flour for food applications, making it a promising functional ingredient for enhancing the nutritional quality of staple products, such as dried noodles.

### 2.2. Noodles Preparation

The ingredients for the dried noodles were prepared and weighed according to the formulation. Four formulas were developed with different substitution levels of WF with *A. ilicifolius* leaf flour (AF): F0 (100% WF), F1 (92% WF: 8% AF), F2 (84% WF: 16% AF), and F3 (76% WF: 24% AF). The eggs were weighed after being beaten, and both WF and *A. ilicifolius* leaf flour were sifted into a container and mixed until evenly distributed. The beaten eggs were added to the dry ingredients, followed by the gradual addition of water while stirring until the mixture was homogeneous. The dough was kneaded for 10–15 min until smooth, as indicated by the absence of dry flour and the formation of a uniform dough ball. The dough was then divided into four portions and sheeted using a noodle press. The sheets were cut into noodle strands and dried in an oven at 180°C for 15 min. To rehydrate the dried noodles, blanching was performed in boiling water (100°C) for the optimum cooking time [13].

### 2.3. Proximate Analysis

The proximate composition was determined using standard gravimetric and Soxhlet methods. Moisture content was analyzed by oven‐drying approximately 1 g of each sample at 105°C until a constant weight was achieved. The ash content was obtained by incinerating the sample in a muffle furnace at 400°C–600°C for 3 h until only mineral residues remained. The acid‐insoluble ash was measured by dissolving the ash in 10% HCl, filtering, incinerating the residue, and weighing the final mass of the residue. The volatile matter was assessed by heating the sample in a muffle furnace at 600°C for 5 min. The fat content was determined using Soxhlet extraction with petroleum ether for 6 h (approximately 15 cycles). Protein content was measured using the micro‐Kjeldahl method, which involved digestion, distillation, and titration with 0.02 N HCl to quantify the nitrogen content, followed by conversion to protein using a factor of 6.25. Carbohydrate content was calculated by difference, subtracting the percentages of moisture, ash, fat, and protein from 100% [14].

### 2.4. Dietary Fiber

Total dietary fiber was determined using the AOAC (1995) multienzyme enzymatic‐gravimetric method. Samples (0.5 g) were sequentially digested with *α*‐amylase, pepsin, and *β*‐amylase under controlled pH and temperature conditions, respectively. Insoluble dietary fiber was collected by filtration, washed with ethanol and acetone, dried at 105°C, cooled in a desiccator, and then weighed. Soluble dietary fiber was precipitated from the filtrate using 95% ethanol, filtered, dried, and weighed. Total dietary fiber was calculated as the sum of the soluble and insoluble fractions.

### 2.5. Antioxidant Activity

Antioxidant activity was measured using the DPPH radical‐scavenging assay, as adapted from [10]. The sample extracts were prepared at different concentrations (100, 200, 300, 400, and 500 ppm). One milliliter of each sample solution was mixed with 1 mL of 0.4 mM DPPH solution and incubated in the dark for 30 min. The mixture was diluted to 5 mL with methanol, and the absorbance was measured at 517 nm using a UV‐Vis spectrophotometer. A blank solution was prepared by mixing 1 mL of DPPH with 4 mL of methanol. Radical scavenging activity (RSA) was expressed as a percentage of inhibition.

The IC_50_ value (the concentration required to inhibit 50% of DPPH radicals) was determined by linear regression of the percentage of inhibition against the sample concentration. Vitamin C was used as a positive control [15].

### 2.6. Organoleptic Test

Organoleptic tests were carried out using two questionnaires: the hedonic test and Just‐About‐Right (JAR). A total of 80 panelists (40 men and 40 women aged 18–30 years) participated in the study. None of the panelists had prior experience in sensory evaluation training and provided written consent. All panelists were gathered to perform sensory testing and filled out the questionnaire via Google Forms. The panelists were asked to taste four samples with different treatments.

In the hedonic test, the panelists were asked to rate each sensory attribute, including color, taste, aroma, texture, and overall attributes, on a 9‐point scale. The scale consisted of “like extremely,” “like very much,” “like moderately,” “like slightly,” “neutral,” “dislike slightly,” “dislike moderately,” “dislike very much”, “dislike extremely.” In JAR test, panelists were asked to rate the sensory attribute with 9‐point scale of the preferred optimal conditions of the color, taste, aroma, and texture [16].

### 2.7. Sensory Panel

The study was reviewed and approved by the Health Research Ethics Commission, Faculty of Medicine, Diponegoro University (Approval Number 208/EC/KEPK/FK‐UNDIP/VIII/2025), and informed consent was obtained from each participant prior to their participation in the study.

### 2.8. Statistical Analysis

Data were analyzed using SPSS Statistics Version 25. Normality was tested using the Kolmogorov–Smirnov test. Normally distributed data were analyzed using one‐way ANOVA followed by Duncan′s test, whereas nonnormally distributed data were analyzed using the Kruskal–Wallis test with Mann–Whitney post hoc comparisons. A 95% confidence level (*p* < 0.05) was used to determine statistical significance.

## 3. Results and Discussion

### 3.1. Nutritional Composition of Dried Noodles

As shown in Table 1, the substitution of WF with *A. ilicifolius* leaf flour significantly influenced the nutritional composition of dried noodles. Moisture content decreased with increasing levels of substitution, from 6.72% in F0 to 1.16% in F3 (*p* < 0.05). Conversely, the ash, protein, and dietary fiber contents increased significantly, with the highest values observed in F3 (ash 3.71%, protein 10.46%, crude fiber 7.91%, and total dietary fiber 9.43%). The carbohydrate and energy values decreased significantly across the formulas, from 76.57% (F0) to 70.91% (F3) and 388.05 to 370.60 kcal, respectively. The fat content did not differ significantly among the formulations. Antioxidant activity improved markedly with increasing substitution, as indicated by the reduction in IC_50_ values from 25,167.79 ppm in F0 to 4324.72 ppm in the F3.

**Table 1:** Nutritional value of four dried noodles formula.

Composition	F0	F1	F2	F3
Moisture (%)	6.719 ± 0.131^a^	2.072 ± 0.299^b^	2.293 ± 0.178^b^	1.160 ± 0.594^c^
Ash (%)	0.679 ± 0.067^a^	2.096 ± 0.385^b^	3.157 ± 0.132^c^	3.714 ± 0.359^d^
Protein (%)	9.740 ± 0.130^a^	9.889 ± 0.509^b^	10.973 ± 0.205^c^	10.460 ± 0.194^c^
Fat (%)	5.710 ± 0.136^a^	5.947 ± 0.196^a^	6.039 ± 0.494^a^	5.849 ± 0.421^a^
Carbohydrate (%)	76.571 ± 0.366^a^	76.346 ± 0.928^a^	71.967 ± 0.375^b^	70.911 ± 0.347^c^
Calories (kcal/100 g)	388 ± 0.354^a^	389 ± 2.480^a^	378 ± 2.851^b^	370 ± 3.669^c^
Crude fiber (%)	0.582 ± 0.142^a^	3.652 ± 0.167^b^	5.571 ± 0.115^c^	7.905 ± 0.566^d^
Insoluble dietary fiber (%)	0.476 ± 0.025^a^	3.664 ± 0.161^b^	6.130 ± 0.152^c^	8.773 ± 0.177^d^
Soluble dietary fiber (%)	0.040 ± 0.000^a^	0.551 ± 0.293^b^	0.604 ± 0.492^c^	0.657 ± 0.021^d^
Total dietary fiber (%)	0.515 ± 0.025^a^	4.215 ± 0.147^b^	6.734 ± 0.187^c^	9.430 ± 0.179^d^
Antioxidant (IC_50_, ppm)	25167.790 ± 503.055^a^	5485.700 ± 139.126^b^	4971.455 ± 120.766^c^	4324.721 ± 124.170^d^

*Note:* Description: F0 (100% WF; 0% AF), F1 (92% WF; 8% AF), F2 (84% WF; 16% AF), and F3 (76% WF; 24% AF). Data were analyzed using one way ANOVA and Duncan′s multiple test. Different superscript letters (a, b, c, d) indicate significant differences in Duncan′s test analysis.

As shown in Table 2, sensory acceptability decreased with increasing substitution levels. The panelists rated F0 (control) significantly higher for color, taste, aroma, texture, and overall acceptability (mean scores between 6.76 and 7.49, corresponding to “like slightly” to “like moderately”). Increasing substitution reduced acceptability, with F3 receiving the lowest scores (“dislike slightly” to “dislike moderately”).

**Table 2:** Organoleptic evaluation of different dried noodles formula using hedonic test.

Parameter	F0	F1	F2	F3
Color	7.49 ± 1.158^a^ like moderately	5.65 ± 1.600^b^ neutral	4.90 ± 1.604^c^ dislike slightly	3.98 ± 1.661^d^ dislike moderately
Taste	6.76 ± 1.460^a^ like slightly	5.58 ± 1.565^b^ neutral	4.66 ± 1.509^c^ dislike slightly	3.90 ± 1.612^d^ dislike moderately
Aroma	6.75 ± 1.547^a^ like slightly	5.46 ± 1.614^b^ neutral	4.80 ± 1.618^c^ dislike slightly	4.10 ± 1.658^d^ dislike slightly
Texture	7.10 ± 1.239^a^ like moderately	5.78 ± 1.567^b^ neutral	4.90 ± 1.666^c^ dislike slightly	4.01 ± 1.563^d^ dislike slightly
Overall	7.06 ± 1.140^a^ like moderately	5.55 ± 1.449^b^ neutral	4.90 ± 1.437^c^ dislike slightly	3.96 ± 1.479^d^ dislike moderately

*Note:* Description: F0 (100% WF; 0% AF), F1 (92% WF; 8% AF), F2 (84% WF; 16% AF), and F3 (76% WF; 24% AF). Data were analyzed using the Kruskal–Wallis difference test and the Mann–Whitney follow‐up test. Different superscript letters (a, b, c, d) indicate significant differences (*p* < 0.05).

As shown in Table 3, the panelists perceived the color to be darker with increasing substitution, shifting from “very bright” (F0) to “slightly darker” (F3). Mangrove taste and aroma intensified at higher substitution levels, with F3 rated as “very bitter” and “slightly grassy.” Texture perception remained consistent across all formulations, rated as “just right.”

**Table 3:** Sensory evaluation of different dried noodles formula using Just‐About‐Right (JAR) method.

Parameter	F0	F1	F2	F3
Bright color	3.25 ± 1.539^a^ very bright	5.18 ± 1.281^b^ just right	6.06 ± 1.205^c^ slightly darker	6.88 ± 1.426^d^ slightly darker
Bitter taste	5.66 ± 1.067^a^ just right	4.85 ± 1.069^b^ slightly bitter	4.41 ± 1.155^c^ slightly bitter	3.96 ± 1.373^d^ very bitter
Grassy aroma	5.05 ± 1.054^a^ just right	4.73 ± 1.043^b^ slightly grassy	4.56 ± 1.311^b^ slightly grassy	4.36 ± 1.701^b^ slightly grassy
Soft texture	5.36 ± .767^a^ just right	5.36 ± .958^a^ just right	5.39 ± .987^a^ just right	5.43 ± 1.230^a^ just right

*Note:* Description: F0 (100% WF; 0% AF), F1 (92% WF; 8% AF), F2 (84% WF; 16% AF), and F3 (76% WF; 24% AF). Data were analyzed using the Kruskal–Wallis difference test and Mann–Whitney follow‐up test. Different superscript letters (a, b, c, d) indicate significant differences (*p* < 0.05).

These findings demonstrate that the partial substitution of WF with *A. ilicifolius* leaf flour improves the nutritional and functional qualities of dried noodles. Moisture content decreased significantly as substitution levels increased, which may be attributed to the higher dry matter content of mangrove leaf flour compared to WF [17]. Lower moisture levels are advantageous for extending the shelf life of dried noodles, as reduced water activity limits microbial growth and enzymatic reactions [18].

The increase in ash content is consistent with the high mineral composition reported in mangrove plants, which are rich in essential elements such as calcium, magnesium, and iron [19]. Protein content also increased modestly, which may be attributed to the additional nitrogenous compounds present in *A. ilicifolius* [20]. Both carbohydrate content and caloric value declined with higher substitution levels, reflecting the displacement of starch‐rich WF by the relatively higher fiber and mineral fractions of *A. ilicifolius*. This reduction is consistent with previous findings where substitution with alternative flours lowered carbohydrate density and overall energy values of staple products [21]. Beyond total carbohydrate content, the nutritional quality of carbohydrates is strongly influenced by starch composition, particularly the ratio of amylose to amylopectin, which plays a key role in determining digestibility and glycemic response. Starch with higher amylose content is generally digested more slowly, resulting in a lower postprandial blood glucose response, whereas amylopectin is more rapidly hydrolyzed and contributes to a higher glycemic impact [22]. Although starch content was not directly measured in this study, the substitution of WF with *A. ilicifolius* leaf flour likely reduces overall starch proportion due to its high dietary fiber content. Dietary fiber has been shown to interfere with enzymatic starch digestion and slow glucose absorption, thereby contributing to improved glycemic control [23]. This suggests that the developed noodles may have a lower glycemic potential compared with conventional wheat‐based noodles. However, further investigation, including starch characterization and in vitro digestibility analysis, is necessary to confirm these potential functional benefits.

A notable improvement was observed in the dietary fiber content, with F3 containing nearly 18 times more fiber than F0. This aligns with previous studies where substitution with mangrove‐derived flours significantly enriched fiber levels in food products [21, 24] Both insoluble and soluble dietary fibers increased significantly across the formulations, with insoluble fiber showing the largest increase. This is expected because mangrove leaves contain a predominance of structural polysaccharides, such as cellulose and hemicellulose, which contribute to the insoluble fiber fraction. Soluble fiber also increased, albeit to a lesser extent, likely due to the presence of pectin and phenolic–polysaccharide complexes in the leaves. Higher fiber intake is associated with a reduced risk of NCDs such as obesity, diabetes, and cardiovascular disorders [1], thus highlighting the potential of *A. ilicifolius* leaf flour in developing functional foods. The incorporation of *A. ilicifolius* leaf flour into dried noodles demonstrates the potential development of functional staple foods with enhanced dietary fiber properties. Functional foods containing dietary fiber are increasingly recognized for their role in reducing the risk of NCDs, particularly obesity, cardiovascular disease, and Type 2 diabetes. Therefore, the developed noodles may provide added health value beyond basic nutrition [1].

### 3.2. Antioxidant Activity

The antioxidant activity improved substantially as the substitution increased, as demonstrated by the lower IC_50_ values. This result is in line with a previous study that reported strong RSA of *A. ilicifolius* leaves due to their high levels of flavonoids, tannins, and polyphenols [3]. Comparable findings have also been documented in other mangrove species such as *Bruguiera gymnorrhiza* [19]. These bioactive compounds contribute to the reduction of oxidative stress and inflammation, suggesting the health‐promoting potential of the developed noodles.

### 3.3. Sensory Evaluation

Sensory evaluation revealed that higher substitution levels negatively affected consumer acceptability. The panelists showed a declining preference for color, taste, aroma, and overall attributes, especially in F2 and F3. Similar challenges were reported where substitution with unconventional flours affected the sensory profile of noodles [13]. The darker color and bitter taste perceived by panelists are likely due to the polyphenolic compounds in *A. ilicifolius*, which, although beneficial to health, can influence palatability [16]. The increased bitterness and color intensity at higher substitution levels are likely due to higher phenolic concentrations, consistent with the findings for leafy vegetable flours. Preprocessing methods, such as brief blanching or the incorporation of natural flavor modulators, could improve the sensory quality of dried noodle samples [25]. Nonetheless, texture acceptability remained stable, indicating that flour substitution did not significantly alter the structural integrity of the noodles.

### 3.4. Cooking Quality Consideration

**Figure 1:**
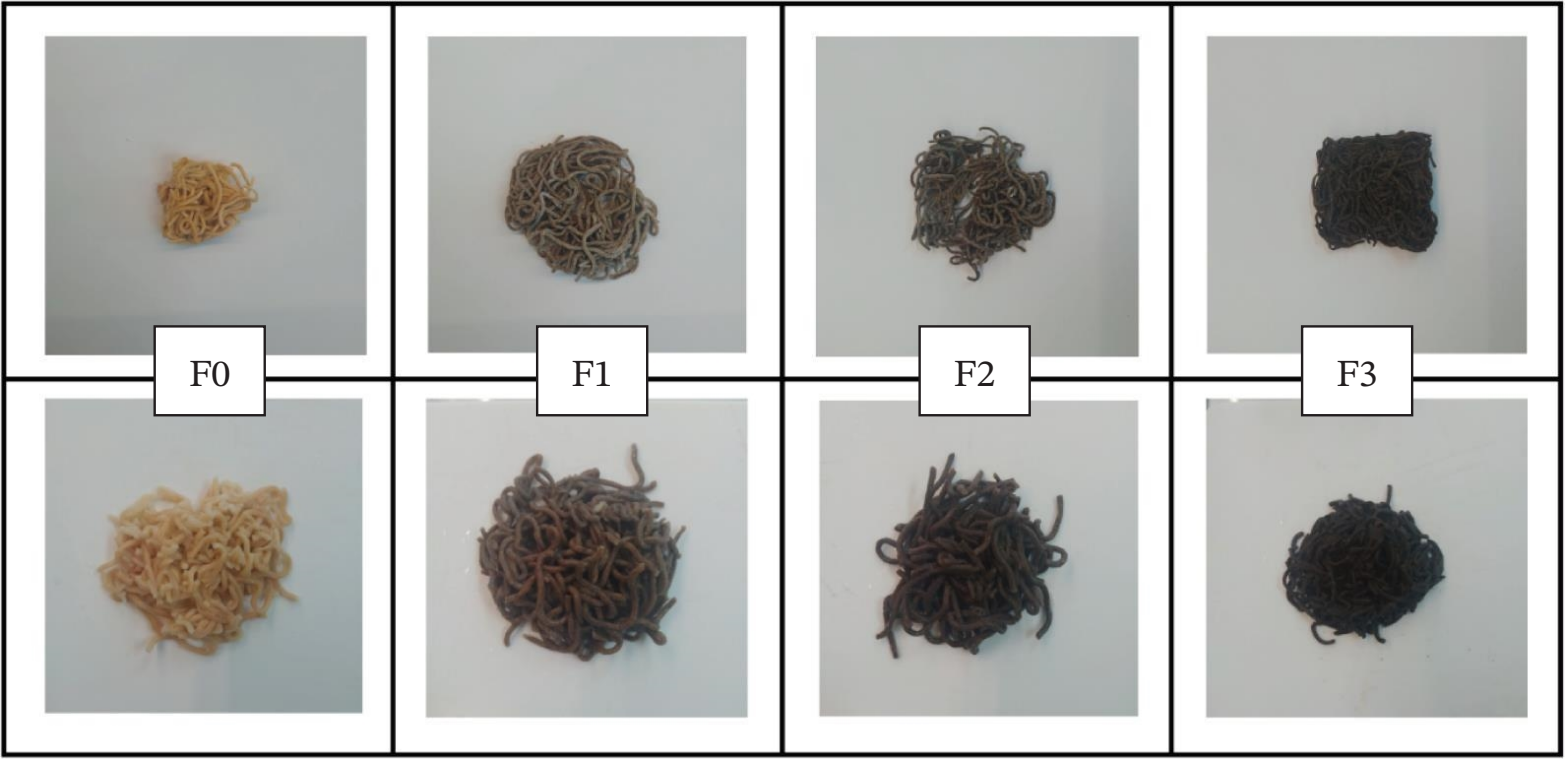
Appearance of dry noodle formulations (top row) and their corresponding boiled products after cooking in water at 100°C (bottom row). From left to right: F0 (control, without *Acanthus ilicifolius* leaf flour), F1, F2, and F3 (with increasing levels of *Acanthus ilicifolius* leaf flour).

In addition to sensory evaluation, cooking quality is one of the important factors influencing taste acceptance of noodles. Although parameters such as optimal cooking time, cooking loss, and breaking rate were not measured in this study, visual observation (Figure 1) indicated that noodles with higher substitution levels tended to be more fragile after cooking. This may be attributed to the reduced gluten network due to partial replacement of WF, which plays a critical role in maintaining noodle elasticity and its structure. Gluten proteins form a continuous network that contributes to the textural stability of cooked noodles, and higher gluten content is associated with improved structural integrity [26]. Conversely, substitution with non‐WFs can weaken this network, leading to increased cooking loss and reduced tensile strength in noodle products [27]. Therefore, future studies should include objective evaluation of cooking properties to better understand the technological performance of *A. ilicifolius* dried noodles.

Overall, these findings suggest that partial substitution, especially at lower levels such as 8% (F1), offers an optimal balance between improved nutritional quality and consumer acceptability of the product. Studies on leafy vegetable flours consistently show a trade‐off between enhanced nutrient content and lower sensory acceptance at higher inclusion levels. For example, moringa flours improved micronutrient profiles but reduced acceptability beyond 10% substitution [28]. Strategies such as partial substitution at lower levels (e.g., F1) or the incorporation of flavor‐masking techniques may balance the nutritional benefits with consumer acceptance.

## 4. Conclusion

Substituting WF with *A. ilicifolius* leaf flour in dried noodles significantly enhanced the nutritional and functional properties, as evidenced by increased ash, protein, dietary fiber, and antioxidant activity, alongside reduced carbohydrate and energy content. In addition, the substitution is likely to influence carbohydrate quality by reducing starch proportion and potentially lowering glycemic response, although this aspect requires further confirmation. Nevertheless, higher substitution levels (≥ 16%) adversely affected sensory attributes, particularly color, taste, and aroma, indicating a trade‐off between nutritional enhancement and consumer acceptability of the product. Although texture remained relatively stable, higher substitution levels also showed a tendency toward reduced structural integrity after cooking, suggesting the importance of evaluating cooking quality parameters such as cooking loss and breaking rate in future studies. Partial substitution at 8% (F1) was identified as the most favorable formulation, providing improved nutritional value while maintaining acceptable sensory characteristics. However, this study has several limitations, including the absence of cooking quality evaluation and starch characterization. Therefore, future research should extend beyond physicochemical and in vitro analyses by evaluating cooking properties, glycemic response, nutrient digestibility, and bioavailability of *A. ilicifolius*‐enriched noodles. In addition, broader consumer acceptance studies involving diverse population groups are recommended to assess market feasibility. Process optimization strategies, such as the pretreatment of leaf flour or flavor‐masking approaches, should also be explored to improve sensory quality at higher substitution levels. These findings support the potential of *A. ilicifolius* leaf flour as a sustainable functional ingredient for the development of nutritionally enhanced staple foods.

## Author Contributions

D.N.A.: conceptualization, supervision, project administration, and preparation of the original draft of the manuscript. K.A.S.: data collection, formal analysis, and visualization. B.N.P.: conducted the investigation, data curation, and provided software support. L.A.B.: contributed to the development of the methodology, validation, and editing of the manuscript. D.N.S.: resources, supervision, and technical guidance throughout the study. D.A.: funding acquisition as well as in the review and editing of the manuscript. B.P.: review, editing, and interpretation of the results. W.H.: validation, critical revision, and gave final approval of the version to be published.

## Funding

This study was supported by the Universitas Diponegoro (10.13039/501100005844, 222‐229/UN7.D2/PP/IV/2025).

## Disclosure

All authors agree to be held accountable for the research presented.

## Conflicts of Interest

The authors declare no conflicts of interest.

## Data Availability

The data that support the findings of this study are available from the corresponding author upon reasonable request.
